# Inequities in Family Planning in Low- and Middle-Income Countries

**DOI:** 10.9745/GHSP-D-23-00070

**Published:** 2023-06-21

**Authors:** John Ross, Karen Hardee, Rebecca Rosenberg, Imelda Zosa-Feranil

**Affiliations:** a Independent demographic consultant, New Paltz, NY, USA.; b Hardee Associates, Arlington, VA, USA.; c Avenir Health, Glastonbury, CT, USA.

## Abstract

**Introduction:**

Inequities in reproductive health are widespread, and periodic surveys can trace trends in inequities to guide policies and program implementation.

**Methods:**

We examined National Composite Index for Family Planning surveys from 2017 and 2021 that assessed inequities in access to 7 contraceptive methods and traced patterns of discrimination involving 5 subgroups in low- and middle-income countries. These surveys use 10–25 informants in each country who are knowledgeable at the national level. Measures are based on questionnaire ratings on a scale of 1–10.

**Results:**

Access to contraceptive methods averages about half of the maximum of 100%, with substantial variation across regions and countries for the score levels. Score profiles are similar among high-scoring and low-scoring countries, suggesting that access to each method reflects common determinants in the nature of each method and the influences acting upon national family planning programs. Access to short-term methods (pill, injectable, and condom) is much better than for long-term methods (sterilization, intrauterine device, and implant). Community-based distribution of contraceptives averages low, as it is not part of some programs. Over time the scores have improved modestly. Correlations imply that better access leads to more contraceptive use. Inequity of use across wealth groups is less where overall equity has improved. Measures of discrimination against youth, unmarried women, postabortion clients, HIV carriers, and different wealth groups indicate a need for additional policies and considerable latitude for stronger actions by providers.

**Conclusions:**

The surveys in 2017 and 2021 demonstrate both progress and deficiencies for equitable access to contraceptive methods, with highly variable results among regions and countries. Much remains to be done to alleviate discriminatory practices against particular subgroups. Equity has improved for access to contraception and contraceptive use, and it can continue to do so with greater attention to policies and practices in national programs.

## Abstract (precis)

Equity has improved for access to contraceptive methods and for measures to lessen discrimination against key subgroups in low- and middle-income countries.

## Teaser

**Key Findings:**

- Equity has improved in all regions through better access to contraception.
- Better access to contraception has led to greater use of the methods, improving equity in contraceptive use.
- Inequities in use across wealth subgroups have been lessened by improved access to methods.
- Discrimination against youth, unmarried persons, HIV carriers, and other subgroups has declined, and more antidiscrimination policies are now in place.

**Key Implications:**

- Management at all levels should move more vigorously to expand access to contraceptive methods, not just individually but in combinations.
- At least 2 long-term and 2 short-term methods are needed everywhere to offer a range of choices that meet the highly variable personal circumstances of both men and women and their aversion to side effects.
- Top officials and budgetary planners may need to address some infrastructure/clinical modifications that may bring subgroups with differential access closer together, thus improving reproductive health equity.
- In addition, actions against subgroup discrimination should be strengthened.

## INTRODUCTION

Health equity has gained increasing attention in recent decades. The World Health Organization (WHO) now lists some 23 health equity indicators^1^ and maintains an extensive database with trends for the indicators in most countries. An earlier analysis examined trends in equity for 18 reproductive health indicators and for gaps between the rich and poor.^2^ Here, we focus more narrowly—to take advantage of recent information in 2 international surveys of 69 low- and middle-income countries—on the topics of discrimination against certain subgroups in the population and equitable access to contraceptive methods.

Our analysis focuses on equitable access to contraceptive methods and discrimination against certain population subgroups.

The literature on international access to contraceptive methods exists primarily in the long series of surveys on national family planning program efforts.^3^ That series, covering most low- and middle-income countries, ran from 1972 through 2014 and was followed by the National Composite Index for Family Planning (NCIFP) series used in this article.^4^ All of these surveys contained similar questions, asking respondents for the proportion of the entire population with “ready and easy access” to each contraceptive method. The latest rounds of the NCIFP for 2017 and 2021 are the basis of this article.

The long series of surveys from 1972 has shown an upward trend in access to contraception across countries, with considerable differences by methods and regions. Countries that began at the lowest levels improved the most, coming closer to the high-scoring countries as they also rose. A review by WHO covering contraceptive use for all methods (not by specific methods) found faster improvements among disadvantaged subgroups and therefore diminishing gaps between them and the more advantaged groups.^5^ Those gains were quite substantial in some countries and subgroups, but serious inequalities persisted. Hosseinpoor and Bergen provide a useful discussion of the WHO work.^6^

A separate series of studies with a similar methodology to the NCIFP—the Maternal and Neonatal Program Effort Index—was devoted to 14 components of maternal and neonatal child health in 49–55 countries, taken over 3 rounds in 1999, 2002, and 2005.^7^ The indicators included the proportion of women with access to family planning services at health centers and district hospitals, postpartum family planning services, antenatal services by pregnant women, and abortions and abortion complications, separately for urban and rural women. The profiles of scores were nearly identical over the 3 rounds, with essentially no improvement in the levels of the scores and all showing rural access to services to be far inferior to those in urban areas. Access to family planning services at health centers and district hospitals ran about 60% of maximum, with much room for improvement.

The literature is fragmentary on the 5 types of discrimination toward the 5 subgroups discussed in this article. Although close estimates of discriminatory practices for a range of countries are not available, discrimination in some countries is officially mandated and commonly observed, as with limits on contraceptive services to unmarried youth. Formal or informal neglect of contraceptive offerings to postabortion clients is present in many countries. Deliberate neglect according to wealth status seems less likely or unintentional; the poorest wealth quintile resides heavily in rural areas where services can be quite limited. Neglect of the top wealth quintile may occur in some programs given their greater self-sufficiency. People living with HIV are often subject to prejudicial treatment within health facilities. In practice, the 5 types of de facto discrimination interact, overlapping by age, marital status, residence, wealth, and HIV status.

United Nations (UN) agencies have taken firm positions against discrimination and inequities. Inadequate policies and practices have long been a concern of such UN agencies as WHO, UN Population Fund, and UNICEF. In particular, the UN’s Sustainable Development Goals, adopted in 2015 to run through 2030, stress commitment to “leaving no one behind,” with goals for deterring discrimination and improving gender equality and sexual and reproductive health and rights, beginning with a target under Goal 5 to “end all forms of discrimination against all women and girls everywhere.” Targets concern whether legal frameworks are in place to deter discrimination based on gender, oppose violence and sexual exploitation against women, and oppose early and forced marriages. Countries are urged to “adopt and strengthen sound policies and enforceable legislation for the promotion of gender equality and the empowerment of all women and girls at all levels.” The final target under Goal 5 goes even further, including an indicator for the proportion of countries with systems to track budget allocations to advance gender equality and female empowerment.^8^

A related UN effort, the United Nations Network on Racial Discrimination and Protection of Minorities, addresses a variety of intersecting issues affecting minority groups with more than 20 UN departments and agencies collaborating.^9^

## METHODOLOGY AND DATA

Data come from the 2017 and 2021 NCIFP rounds, covering 69 countries common to both years. In addition, data on contraceptive use are from an extensive compilation of national surveys by the UN.^10^ Among the latest national surveys for the 69 countries included in the NCIFP in 2021, 53 were taken from 2016 onward, 12 in the previous 5 years for 2011–2015, and 4 for 2005–2010.

The NCIFP surveys cover a broad range of reproductive health indicators, organized under the 5 dimensions of strategy, data management, quality, equity, and accountability. This article focuses on the equity dimension (Box). Under the headings “policies” and “providers,” there were 5 questions each concerning 5 subgroups, namely youth, unmarried women, wealth status, postabortion, and HIV status. This was followed by an item for community-based distribution (CBD) of contraceptives and 7 questions for access to 7 contraceptive methods. Access to the 4 long-term methods was averaged as the long-term method score, and the 3 short-term methods were averaged as the short-term method score. Detailed questions for the equity score are included in Supplement 1.

BOX Equity-Related Items in the National Composite Index for Family Planning

1. Are there policies in place to prevent discrimination toward special subgroups?^a^
2. To what extent do service providers discriminate against special subgroups?^a^
3. Extent to which areas of the country are not easily served by clinics or other service delivery points are covered by community-based distribution programs for distribution of contraceptives (especially in rural areas)
4. Extent to which the entire population has ready and easy access to long-term methods (female sterilization, male sterilization, intrauterine devices, and implants)
5. Extent to which the entire population has ready and easy access to short-term methods (condoms, pills, and injectables)

^a^Subgroups include youth, unmarried women, wealth status, postabortion, and HIV status.

Questionnaires were completed in each country by 10–25 expert respondents familiar with the national picture, drawn from various institutions and professional specialties. For each country, a study manager was selected and trained to identify appropriate respondents, introduce the questionnaire, obtain the responses, and send them in for central analysis. This approach—used in the Family Planning Effort Index, Maternal and Neonatal Program Effort Index, and NCIFP surveys—makes possible a set of measures on numerous indicators for many countries at a single point in time and at reasonably low cost. It also allows for expert judgment to produce estimates on variables for which no comprehensive data exist.

Because the ratings depend upon the judgments of observers in each country, questions have been raised as to possible biases favoring the national program that might heighten the correlations between program efforts and contraceptive use. However, negative biases against the program are also possible. To help protect against this bias, multiple types of respondents were always used to include some of those close to the program and others in university departments, local agencies, or donor agencies. Ratings were scanned to spot improbable outliers, with follow-up inquiries. As a test of the objectivity of the ratings, a special study in Kenya and Bangladesh was conducted to gather direct, empirical measures on some indicators to compare the results to the observer ratings, which found substantial agreement between them.^11^ Illustrative scores that were examined included statements by national leaders regarding the national program, implementing assistance by the civil bureaucracy, multi-ministry involvements, import laws, and budgets. Although a useful test, it was conducted in only 2 countries, with some disagreement between them. Over the years, a further check has been the overall consistency of scoring across countries, both overall and for subscores.

Scoring in the questionnaire ran on a scale from 1 to 10, given as a percentage (e.g., a score of 6 is presented at 60%). For uniformity, all scores were converted so that a high score always represents a favorable outcome (e.g., greater equity).

Some results are given separately for 6 geographic regions: Asia; Eastern Europe and Central Asia (EECA), Latin America and the Caribbean (LAC), Middle East and North Africa (MENA), Eastern and Southern sub-Saharan Africa (ESA), and Central and Western sub-Saharan Africa (CWA). Supplement 2 provides a full list of the 69 countries by region covered in the NCIFP surveys.

Methods include crosstabulations, correlations, and graphical presentations. Regional and total averages are unweighted, giving every country equal importance. With population weights, China and India, for example, would disproportionally control the averages for Asia and for all countries.

## RESULTS

We report on the following equity patterns in the 2021 NCIFP across the 69 countries: policies against discrimination and discriminatory practices by providers regarding 5 subgroups, presence of CBD programs where needed, and access for 7 contraceptive methods. Regional patterns and selected time trends are discussed. We also present: (1) individual country data for a summary of equity indicators; (2) correlations between equity and contraceptive use, by method; and (3) disparities in equity according to wealth quintiles for a subset of countries.

### Discrimination

Equity is compromised when particular subgroups in the population are discriminated against. Two questions were directed to that issue. The first question concerned national policies: “Are there policies in place to prevent discrimination towards special subgroups?” Five subgroups were listed: youth, unmarried women, wealth difference, postabortion, and HIV status. Policy strength was scored for each: 1=nonexistent and 10=strong policies.

The second question related to the actions of providers: “To what extent do service providers discriminate against special subgroups?” Again, the 5 subgroups were listed, each scored with 1=providers discriminate extensively and 10=providers do not discriminate. In presenting the findings, high scores for providers indicate nondiscrimination. A preliminary note is that “discrimination” may be deliberate, or it may mask unintentional deficiencies across subgroups due to difficulties in serving remote areas or in countering public ignorance of services. These overlap in accounting for lower contraceptive use among subgroups.

#### Outcomes for Policies and Providers

**FIGURE 1:**
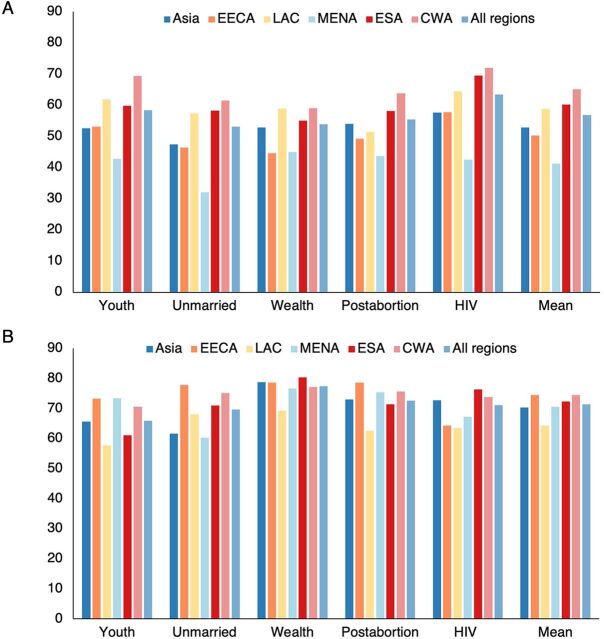
Average Scores for (A) Policies Against Discrimination and (B) Provider Practices Against Discrimination, by Subgroup and Regions Abbreviations: CWA, Central and Western sub-Saharan Africa; EECA, Eastern Europe and Central Asia; ESA, Eastern and Southern sub-Saharan Africa; LAC, Latin America and the Caribbean; MENA, Middle East and North Africa.

A curious finding is that the scores are much lower for policies than for provider practices (Table 1 and Figure 1). Across all 5 groups and all regions, the average policy score of 56.9% falls well below the much higher average of 71.5% for practices (comparing the 2 panels, rightmost bars, in Figure 1). However, the question only asked whether policies were in place to prevent discrimination. A failure to establish such policies yielded a low score. Some countries may have established antidiscrimination policies and deserved high scores, but in the overall regional and total scores, they are averaged with the countries that took little or no action.

**TABLE 1.** Average Scores for Policies Against Discrimination and Provider Practices Against Discrimination, by Subgroup and Region

	**Youth, %**	**Unmarried, %**	**Wealth, %**	**Postabortion, %**	**HIV, %**	**Mean, %**
Policies						
Asia	52.6	47.5	52.9	54.0	57.7	52.9
EECA	53.2	46.4	44.6	49.3	57.7	50.2
LAC	61.9	57.4	58.9	51.5	64.5	58.9
MENA	42.8	32.0	45.0	43.8	42.5	41.2
ESA	59.8	58.2	55.1	58.2	69.5	60.2
CWA	69.3	61.6	59.1	63.8	72.0	65.2
All regions	58.5	53.2	53.9	55.4	63.5	56.9
Providers						
Asia	65.6	61.7	78.8	73.1	72.7	70.4
EECA	73.3	77.9	78.6	78.6	64.3	74.6
LAC	57.7	68.1	69.3	62.6	63.5	64.3
MENA	73.4	60.3	76.7	75.4	67.2	70.6
ESA	61.2	71.1	80.4	71.4	76.4	72.4
CWA	70.6	75.2	77.1	75.7	73.8	74.5
All regions	66.0	69.7	77.5	72.7	71.2	71.5

Abbreviations: CWA, Central and Western sub-Saharan Africa; EECA, Eastern Europe and Central Asia; ESA, East and Southern sub-Saharan Africa; LAC, Latin America and the Caribbean, MENA, Middle East and North Africa.

Policies to avoid discrimination score lower than provider efforts to avoid discrimination in practice.

The regional rankings are quite different between policies and practices related to the subgroups. The mean values show that for policies, the MENA region is consistently the lowest scorer, at 41.2%, with the fewest policies opposing discrimination. However, for providers, the LAC region scores lowest at 64.3%. The highest scores for policies are for the ESA and CWA regions, with the LAC region close behind and the others reflecting some regional irregularity. For provider practices towards the subgroups, most regional means are clustered between 70% and 74%, with less irregularity.

#### Ratings by the 5 Subgroups

For policies, the highest average rating is 63.5% for HIV status (Table 1, All regions row). That is consistent with the strong attention to stigma and discrimination common in HIV/AIDS programming. Ratings for the other subgroups are not far apart, with scores in the low to mid-50s. However, considerable variation exists within particular regions.

For provider practices, the subgroup differences are more marked, from a low of 66.0% for youth to the most favorable of 77.5% for wealth groups, signifying rather little discrimination by providers according to the wealth status of clients and more against youth. The other 3 groups (unmarried women, postabortion women, and wealth status) are not much different, ranging only from 69.7% to only 72.7%. Again, particular regions have their own patterns, discriminating to lesser or greater extents across the subgroups.

We previously noted here the surprisingly much lower scores for policies to promote nondiscrimination than for providers giving nondiscriminatory care. However, the gap between them differs widely by subgroup. The gap ranges from only 8 points for youth and HIV to a high of 23.6 points for wealth groups due to the exceptionally high provider score of 77.5%, which signals rather little discrimination by providers according to the wealth of clients. Close to that is the gap of 17.3 for postabortion clients, reflecting the high provider score of 72.7%. That says that providers discriminate rather little regarding postabortion services. Note that the policy score for discrimination according to HIV status is by far the highest policy rating, hence the small gap with provider practices.

The policy measures related to the 5 subgroups are highly intercorrelated, meaning that respondents in different countries were prone to give similar scores, whether low or high, to the measures related to the 5 subgroups (data not shown). The same was true of the measures for discrimination by providers, with high correlations among the 5 subgroups. However, policies against discrimination and providers’ discriminatory practices do not correlate well with each other and so are measuring somewhat different things.

### Community-Based Distribution

The NCIFP study asked for a rating of CBD programs with the following:

*Extent to which areas of the country not easily serviced by clinics or other service points are covered by CBD programs for distribution of contraceptives (especially rural areas). (1=nonexistent; 10=extremely high coverage)*

The resulting scores were low, averaging only 49.0% for all regions but across a very wide range, from 35.0% in EECA to nearly double that (59.4%) in CWA (data not shown). LAC was also low at 40.7%; the others were clustered in between: MENA at 48.7%, ESA at 49.3%, and Asia at 52.6%.

The reasons for these differences probably reflect the nature of the national family programs, which typically embrace any existing dedicated CBD efforts. For example, EECA is composed of the former republics of the Union of Soviet Socialist Republics in Central Asia and the Caucasus, where programs did not deploy rural CBD workers to the same extent as in other countries. The same may be said of the LAC region, where clinic services are more prevalent. CBD efforts score higher in sub-Saharan Africa (SSA), but the large score difference between CWA (59.4%) and ESA (49.3%) calls for further investigation.

### Access to Contraceptive Methods

The NCIFP survey asked respondents to rate “the extent to which the entire population has ready and easy access” to each of 7 contraceptive methods (long-term methods: female sterilization, male sterilization, the intrauterine device [IUD], and the implant; short-term methods: the condom, pill, and injectable).

**FIGURE 2:**
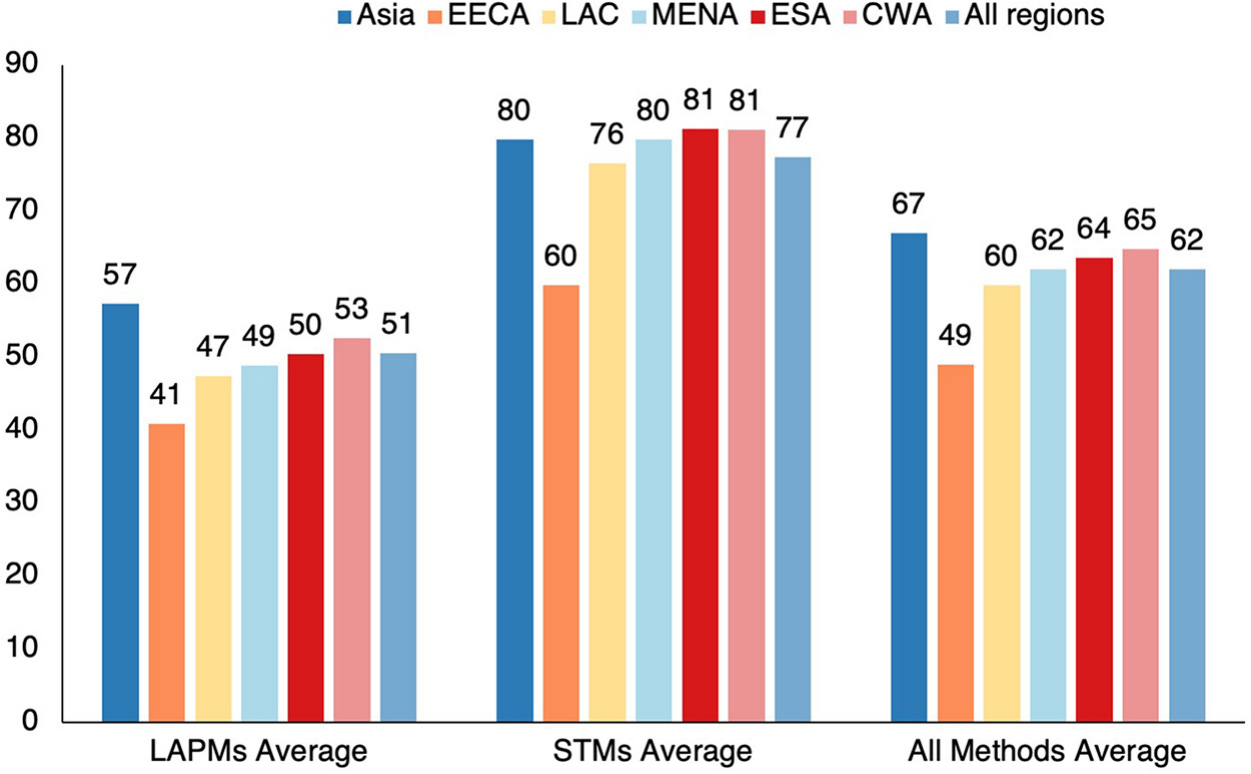
Access to Contraceptive Methods, by Type and Region Abbreviations: CWA, Central and Western sub-Saharan Africa; EECA, Eastern Europe and Central Asia; ESA, Eastern and Southern sub-Saharan Africa; LAC, Latin America and the Caribbean; LAPM, long-acting and permanent method; MENA, Middle East and North Africa; STM, short-term method.

Access to short-term methods is far better than access to long-term methods, which are mainly clinical (Figure 2). That is true in every region by large margins. Overall, the average is 77% for short-term methods, 26 points above a low 51% for long-term methods. That agrees closely with results from the 2017 NCIFP: 74.4% for short-term methods and 44.2% for long-term methods, although access to both types of methods improved between the 2 survey years (data not shown).

**FIGURE 3:**
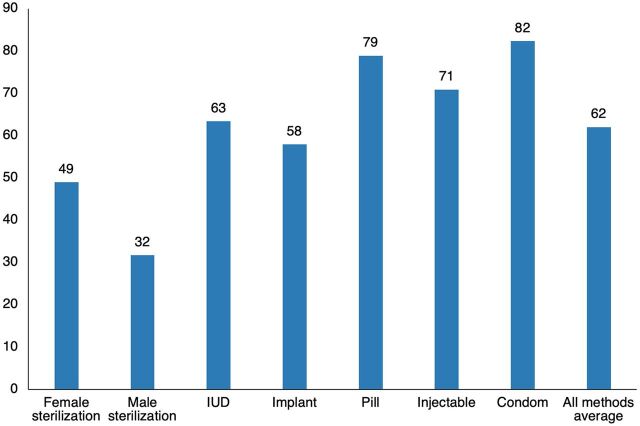
Access to Each Contraceptive Method, All-Country Averages Abbreviation: IUD, intrauterine device.

Around the average, access to the individual methods falls across a wide range, from a low of only 32% for male sterilization to the high of 82% for the condom, with the pill close behind at 79% (Figure 3). The injectable ranks next, followed by all of the long-term methods.

Regions differ substantially in the access to the 7 methods (Table 2). In Asia, access is especially high for both male and female sterilization (46.9% and 60.1%, respectively). The other regions vary little—around 44%–49% for female sterilization and much lower figures for male sterilization, with more variability. For the IUD, access is high, mainly in EECA and MENA (72.8% and 74.4%, respectively); in both regions, the IUD has been popular for decades. Asia also scores high on access to the IUD at 68.0%. Access to the implant and injectable stand out in both ESA and CWA regions, with a fairly long history of their implementation in numerous SSA countries. Access levels for the pill and the condom show less regional variation, and they fall at higher levels than for the other methods. The all-region average shows them to rank highest at 78.9% and 82.4%, by large margins from the other methods.

**TABLE 2.** Percentage of Population With Ready Access to Each Contraceptive Method, by Region

	**Female Sterilization, %**	**Male Sterilization, %**	**IUD, %**	**Implant, %**	**LAPMs Average, %**	**Pill, %**	**Injectable, %**	**Condom, %**	**STMs, Average, %**	**All Methods, Average, %**
Asia	60.1	46.9	68.0	54.4	57.3	82.3	73.0	83.9	79.7	66.9
EECA	47.2	21.3	72.8	22.2	40.9	63.3	40.4	75.8	59.8	49.0
LAC	46.9	29.4	55.2	57.8	47.3	76.2	74.2	79.1	76.5	59.8
MENA	45.7	19.9	74.4	55.1	48.8	87.7	70.5	81.0	79.7	62.0
ESA	43.7	32.3	57.3	68.3	50.4	81.7	78.3	83.6	81.2	63.6
CWA	47.9	28.8	61.6	71.9	52.5	80.6	77.0	85.9	81.2	64.8
All regions	49.0	31.8	63.4	57.9	50.5	78.9	70.9	82.4	77.4	62.0

Abbreviations: CWA, Central and Western sub-Saharan Africa; EECA, Eastern Europe and Central Asia; ESA, East and Southern sub-Saharan Africa; LAC, Latin America and the Caribbean, LAPM, long-acting and permanent method, MENA, Middle East and North Africa; STM, short-term method.

What explains these differences in access? For national programs and the commercial sector, it is easier to deploy condoms and pills than to set up facilities for sterilization and the IUD across most of the country. There is also a kind of feedback between the actual use of a method and its access. The unpopularity of male sterilization encourages its neglect by providers, whereas the early popularity of the injectable in East Africa led to an increase in services and donor support for it. In Latin America, the early popularity of female sterilization led to greater access for it, encouraged by the appearance of simple laparoscopic technology. On the other hand, the cultural adversity to female sterilization in the MENA region increased attention to the IUD, and the private medical sector helped ensure its widespread access.

Regions vary in the access they give to different contraceptive methods, with the exception that the condom scores well everywhere.

### Time Trends

Time trends for the equity indicators can be traced over the period of 4 years between the 2017 and 2021 rounds of the NCIFP survey for the 62 countries with data for both years. The changes for policies and providers were relatively minor—the former rose by 1.9% and the latter declined by 0.5% (Table 3). However, the scores rose substantially for CBD (15.2%) and long-term methods (14.2%) though much less so for short-term methods (3.4%). The changes between 2017 and 2021 were reflected in the percentage of countries showing improved scores: only about half of countries did so for policies and providers (56.5% and 48.4%, respectively) but considerably more for CBD and long-term methods (72.6% and 77.4%, respectively) and much less for short-term methods (56.5%).

**TABLE 3.** Average Equity Scores in 2017 and 2021 Rounds of NCIFP Survey

	**Policies, %**	**Providers, %**	**CBD, %**	**LAPMs, %**	**STMs, %**
2017	55.5	71.7	42.2	44.2	74.7
2021	56.6	71.3	48.7	50.4	77.2
Change (%)	1.1 (1.9)	−0.3 (−0.5)	6.4 (15.2)	6.3 (14.2)	2.5 (3.4)
How many countries showed an improvement?					
No. (%)	35 (56.5)	30 (48.4)	45 (72.6)	48 (77.4)	35 (56.5)

Abbreviations: CBD, community-based distribution; NCIFP, National Composite Index for Family Planning; STM, short-term method; LAPM, long-acting and permanent method.

The trend for the overall equity score can serve as another perspective on past improvements while giving the average scores for both years. Pertaining to the 62 countries with data in both rounds, Table 4 shows that the average score rose by a rather impressive 7 points (about 12%) over the 4 years. That is a favorable outcome, but it varied by region. The smallest gain was in ESA, but it started from the highest level in 2017. CWA rose by a remarkable 10.4 points, starting from a relatively low level. The gain in MENA was 9.4 points, starting from a very low level in 2017. The other 3 regions had gains between 5.5 and 8.2 points.

**TABLE 4.** Changes in the Average Equity Score, by Region

	**2017, %**	**2021, %**	**Increase, %**
Asia	53.7	61.1	7.4
EECA	50.5	56.1	5.5
LAC	52.2	60.4	8.2
MENA	46.9	56.3	9.4
ESA	60.9	64.2	3.3
CWA	56.9	67.2	10.4
Total	55.1	62.0	6.9

Abbreviations: CWA, Central and Western sub-Saharan Africa; EECA, Eastern Europe and Central Asia; ESA, East and Southern sub-Saharan Africa; LAC, Latin America and the Caribbean, MENA, Middle East and North Africa.

### Inequities in Contraceptive Use in Relation to Access

To what extent does better equity in access to contraception translate to more contraceptive use? The correlations between access and use by method are suggestive though not causal (Table 5). (Data on use for the 69 countries are from the 2022 UN compilation of national surveys cited previously.)

**TABLE 5.** Correlations Between Access and Contraceptive Use

	**All** **Countries, r**	**Non-SSA, r**	**SSA, r**
Female sterilization	0.25	0.26	0.15
Male sterilization	0.34	0.40	0.12
IUD	0.63	0.71	0.55
Implant	0.46	0.37	0.28
Pill	0.31	0.43	0.22
Injectable	−0.43	−0.41	−0.09
Condom	0.11	0.06	0.09
Short-term methods	0.23	0.44	0.16
Long-term methods	0.13	0.12	0.10
All methods	0.22	0.41	0.25

Abbreviations: IUD, intrauterine device; SSA, sub-Saharan Africa.

Although the correlations are not large, most of them are in the expected direction. The IUD stands out as showing the closest dependence of use upon access, by the highest “r” value of 0.63. Next in order, well below the IUD, are the implant, the pill, female sterilization, and condom. The injectable correlation is an unexpected negative relation, possibly an artifact, as the scattergram for the data points shows a few outliers where access is rated very low but use is very high, with the rest of the countries in a neutral to positive pattern between access and use.

Better access led to greater contraceptive use for nearly all methods.

Regions outside of SSA show higher correlations between access and use for every method except the condom, and the difference is quite substantial for short-term methods and for all methods though not for long-term methods (final rows of Table 5). In scattergrams, the access and use levels for the regions outside of SSA both run at higher levels than in SSA while also showing less scatter around the least square lines.

### Access in Individual Countries by Region

**FIGURE 4:**
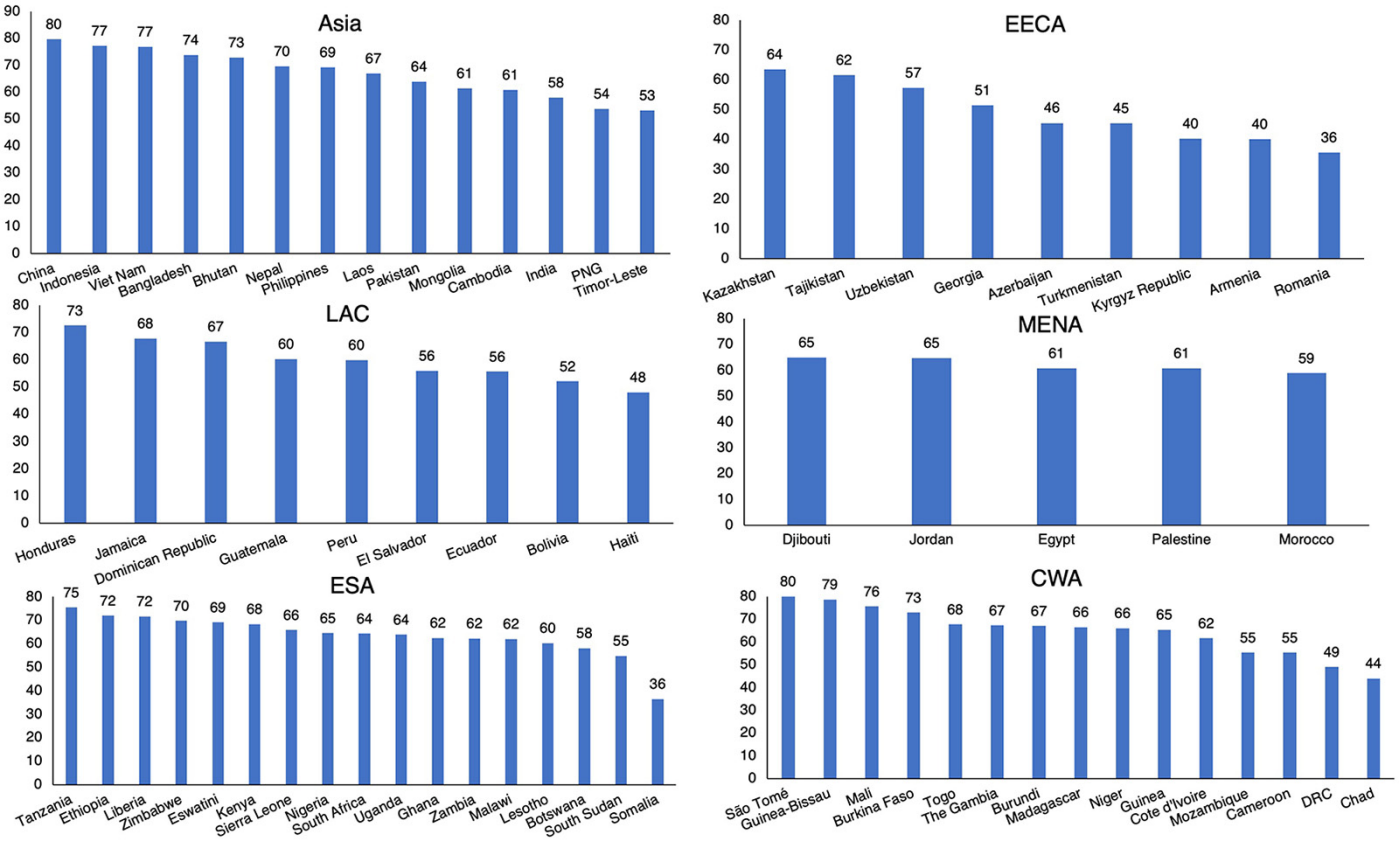
Mean Access for 7 Contraceptive Methods, by Region Abbreviations: CWA, Central and Western sub-Saharan Africa; DRC, Democratic Republic of the Congo; EECA, Eastern Europe and Central Asia; ESA, Eastern and Southern sub-Saharan Africa; LAC, Latin America and the Caribbean; MENA, Middle East and North Africa; PNG, Papua New Guinea.

Within each region, countries differ substantially in access and, therefore, in equity. Using the mean of the access ratings across the 7 contraceptive methods, Figure 4 displays the countries in order in each region. The regional means accompanying Figure 4 are Asia (64.1%), EECA (53.6%), LAC (57.5%), MENA (59.0%), ESA (64.6%), and CWA (68.2%). Nearly all countries show scores above the 50% level, but fewer do so above the two-thirds (67%) line. However, half do so in Asia and a third in both ESA and CWA region but only a fifth in LAC and none in EECA or MENA. Only a few countries score below 50%, but in EECA, 5 of 9 do so, in addition to 1 each in LAC and ESA and 3 in CWA.

This is a regional picture of different central tendencies, different spreads from high to low scores, and differences in the highest scores, from China as highest to Romania and Somalia as lowest. Figure 4 demonstrates that access to contraception has room to improve in many countries.

**FIGURE 5:**
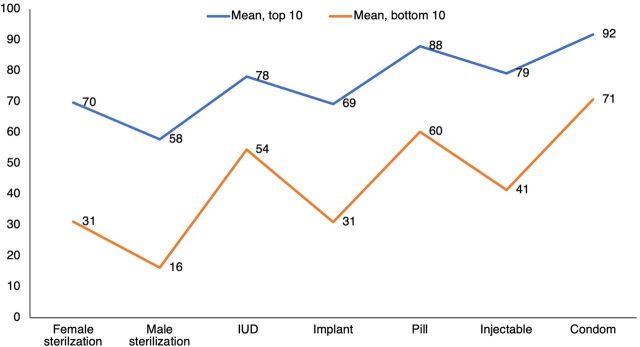
Profiles of Access to 7 Contraceptive Methods: Top 10 and Bottom 10 Countries Abbreviation: IUD, intrauterine device.

It is especially interesting that despite dissimilar levels of access, the countries show similar profiles across the 7 contraceptive methods. Figure 5 compares the 10 highest-scoring and 10 lowest-scoring countries by their access to the methods. First, the greatly different levels attest to the broad range of access among the countries. The average across the top line in the figure is 77% but only 44% for the bottom line—a difference of 33 points. Second, and quite remarkable, is the similarity between the 2 profiles. Regardless of levels, the high-scoring and low-scoring countries differ little in the relative preference they give to the various methods. That occurs despite the dissimilar regional mixes of the 2 sets of countries and the contrasts in country circumstances and programs. To an extent, the low-scoring countries are simply acting similarly but doing everything with less effort.

### Equity Disparities in Contraceptive Use by Wealth Quintiles

The NCIFP obtained ratings on equity levels, which leaves open the question of disparities within a country around the average levels. As the levels rise, do subgroups in the population show greater similarity (i.e., less inequity and smaller disparities)? The NCIFP study lacks data on access disparities as such, but with data from Demographic and Health Surveys, we can determine whether disparities in contraceptive use decline as average access rises. The analyses show that better access leads to more use; here, we ask whether disparities in use across wealth quintiles also shrink with better access.

It is better to explore this with the 2017 round than the 2021 round because few Demographic and Health Surveys (only 22) occurred in years close to 2021 for any of the 69 countries in the NCIFP. For 2017, there were 33 countries that had surveys that occurred closely either before or after 2017 (within 6 years before or any time after). About two-thirds of these countries are in SSA, divided about equally between ESA and CWA. The analysis here poses the degree of disparity in contraceptive use against the average access level by method.

If a higher score on access produces greater uniformity of use across wealth quintiles, we would expect that where access is good, use would be more similar across wealth quintiles because the poor would have increased their use more than the rich, who were already at higher levels. Disparity here is measured by the average deviation (AD) method.^12^ The lower the AD score, the less the disparity, so a negative relationship should emerge between disparity and access.

The AD measures the degree of disagreement in contraceptive use across the 5 quintiles. It takes the differences from the mean, whether positive or negative, adds them, and then averages them. If all 5 quintiles are equal in terms of use, then there are no differences, and the score is zero. At the other extreme, where 1 quintile has 100% of use and the others have zero, the score can reach 32. In between, most countries show quintile differences in the middle range. None are at zero because there is some difference everywhere in use among wealth quintiles.

In fact, better access does accompany less disparity in use of methods by wealth quintiles for 5 of the 6 contraceptive methods (Table 6). As access improves, the disparity across wealth groups declines, reducing inequities, as suggested by the negative correlations shown. The relationship is strongest for female sterilization, IUD, and condom and somewhat weaker for the implant and pill. For the injectable, the association is slightly positive but at a minimal level. (Male sterilization is omitted due to many missing values for its use.)

**TABLE 6.** Correlations Between Contraceptive Access and Disparity Across Wealth Quintiles

	**r**
Female sterilization	−0.40
IUD	−0.30
Implant	−0.23
Pill	−0.17
Injectable	0.08
Condom	−0.41
STM	−0.18
LTM	−0.31
All methods	−0.35

Abbreviations: IUD, intrauterine device; LTM, long-term method; STM, short-term method.

A closely related question is whether disparities in contraceptive use diminish as total prevalence of use rises. If they do, that reflects the chain of influences from better access to increased use and on to less inequity for both access and total use. We would expect that where total prevalence is high, the use of several methods would necessarily be high, and they would not be too far apart across wealth quintiles. That would make for less disparity and more equity.

**FIGURE 6:**
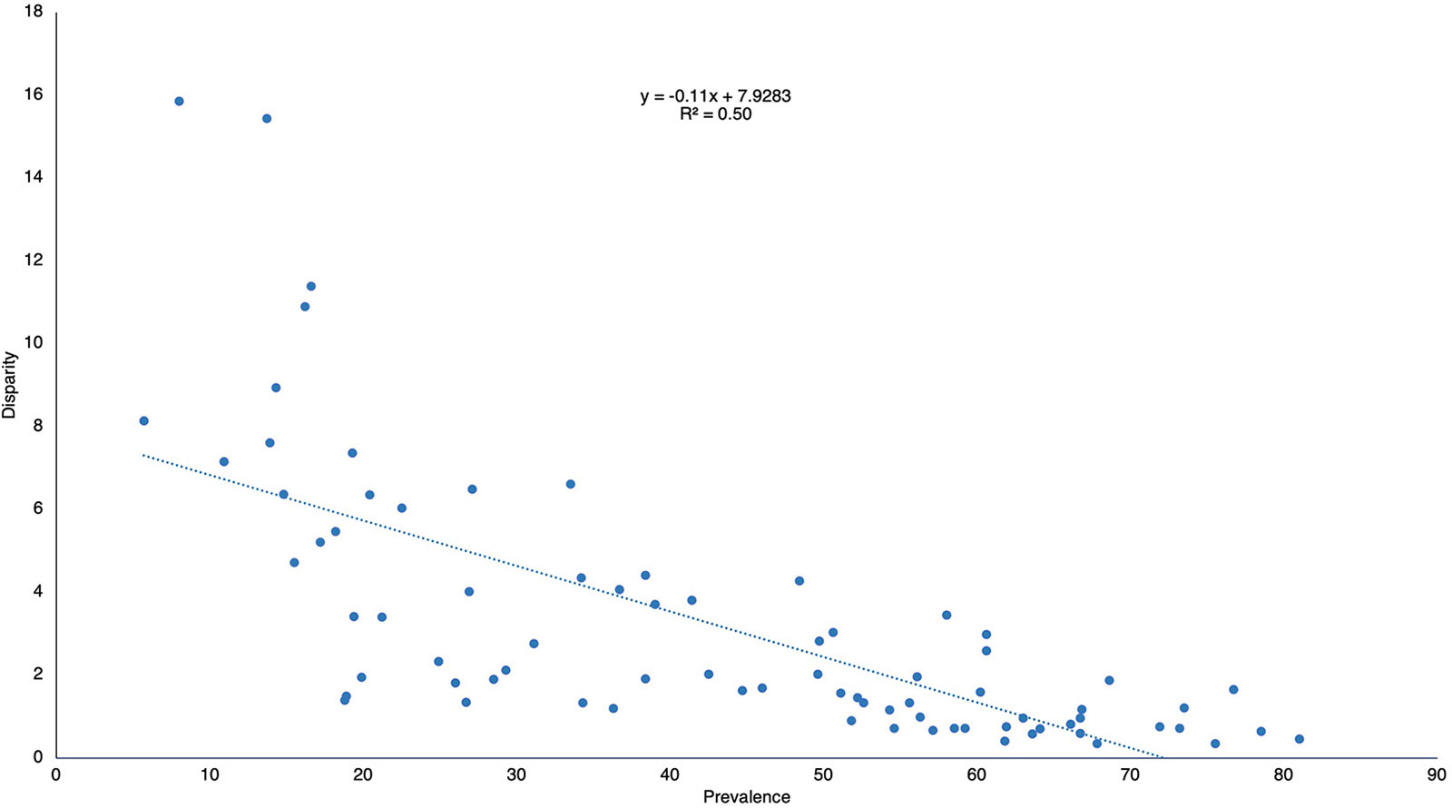
Relation of Disparity Across Contraceptive Methods by Total Prevalence

The expectation is firmly confirmed: disparity (inequity) in total contraceptive use across quintiles declines as total use increases (Figure 6). There are roughly 3 stages: at the extreme left, for countries with very low prevalence, many depend chiefly on 1 or 2 methods, with the rest near zero, and that makes for high disparity. Next, there is a gradual move by countries to deploy more methods, raising their shares of total use, hence less disparity. Finally, there is the crowding that occurs at the right. At that point, a country simply cannot have high prevalence unless most methods are at high and fairly even levels of use across wealth quintiles, hence less disparity and inequity.

Equity improves across wealth groups with higher access scores and greater contraceptive use.

## DISCUSSION

The NCIFP surveys of 2017 and 2021 show continuing shortfalls in access to contraception and in efforts to combat discrimination against key subgroups in the population. However, advances in access to contraception have led to its greater use and to reduced inequities in use according to wealth status. Access is much greater for short-term methods than for long-term methods, but over time equity measures have improved more for long-term methods and for CBD distribution. The range of scores is very large across regions and countries, creating much potential for the low-scoring countries to examine and learn from policies and practices in the high-scoring countries in their regions.

The uneven levels of access across the 7 contraceptive methods are important to note. Certain methods are inherently less likely to win widespread access than others; the extreme examples are male sterilization at the low end and the condom at the high end. For most methods, the regions vary considerably in access levels, reflecting historical and cultural disparities as seen, for example, by the prominence of the IUD in the MENA region and female sterilization in Latin America.

The persistence of access differences by method raises the question of whether access can only be improved so much. How high can the access scores go against the maximum of universal availability? If, for example, the achievements of the top 5% of countries on each method are taken as “par,” then that sets a modified standard against which to score the other countries. It would raise the current scores in comparison to what might reasonably be expected of them in the future.

A further constraint on prevalence levels is women’s own preferences as they interact with program priorities. Historically, the pill and implant found wide adoption in some SSA countries, as female sterilization did in Latin America. Statistical measures of unmet need correspond only roughly with women’s reports as to their own intention to use a method, with each group lacking members in the other one.

Access measures are generally focused on single methods, whereas women need easy access to multiple methods as their needs evolve over time. In each country, ratings of access should be improved to show the proportions of women with access to at least 2 short-term methods and at least 2 long-term methods.

Ratings of access should be improved to show the proportions of women with access to at least 2 short-term methods and at least 2 long-term methods.

### Limitations

Limitations to the findings here include those that attend expert ratings by respondents, with their possible biases either high or low, as discussed in the text. Measurement errors of many types are a hazard, as in all survey research, including questionnaire design and interviewer selections. All survey results are subject to problems of both reliability and validity and to misinterpretations by analysts. The use of repeat surveys, as in this report, is helpful in identifying some shortcomings.

## CONCLUSION

Discrimination against vulnerable subgroups is regrettable on numerous grounds, including human rights, as stressed continuously by the UN and other agencies. Policies against discrimination are inadequate in many countries, and provider practices fall short. Discrimination constrains access to contraceptive methods by the affected groups, raising the rates of unwanted pregnancies and births. Improved access levels for vulnerable or marginalized groups can help avoid the human costs involved and can be achieved through closer attention to national policies and to field practices.

## Supplementary Material

GHSP-D-23-00070-supplements.pdf

## Notes

**Peer Reviewed**

**First published online:** June 20, 2023.
