# Supplementary material for: Inequities in Family Planning in Low- and Middle-Income Countries
Source: Glob Health Sci Pract. 2023 Jun 21;11(3):e2300070. doi: 10.9745/GHSP-D-23-00070 (PMC10285719; doi:10.9745/GHSP-D-23-00070)
Supplement: GHSP-D-23-00070-supplements.pdf [file GHSP-D-23-00070-supplements.pdf]

Supplement to: Ross J, Hardee K, Rosenberg R, Zosa-Feranil I. Inequities in family planning in low- and middle-income countries. *Glob Health Sci Pract*. 2023;11(3):e2300070. https://doi.org/10.9745/GHSP-D-23-00070

Supplement 1. Detailed Questions for Equity Score

Are there policies in place to prevent discrimination toward special subgroups?

**Policies**	Policies to prevent discrimination: Youth<br>Policies to prevent discrimination: Unmarried Women<br>Policies to prevent discrimination: Wealth Status<br>Policies to prevent discrimination: Post-abortion<br>Policies to prevent discrimination: HIV Status

To what extent do service providers discriminate against special subgroups?

**Providers**	Providers discriminate: Youth<br>Providers discriminate: Unmarried Women<br>Providers discriminate: Wealth Status<br>Providers discriminate: Postabortion<br>Providers discriminate: HIV Status
**Community-based distribution (CBD)**	Extent to which areas of the country not easily serviced by clinics or other service points are covered by CBD programs for distribution of contraceptives (especially rural areas).
**Access**	Extent to which the entire population has ready access to each of 4 long-acting and permanent methods (LTMs) and to 3 short term methods (STMS)<br>**Access to LTMs**<br>Access to: Female Sterilization<br>Access to: Male Sterilization<br>Access to: IUDs<br>Access to: Implants<br>**Access to STMs**<br>Access to: Condoms<br>Access to: Pills<br>Access to: Injectables

Supplement to: Ross J, Hardee K, Rosenberg R, Zosa-Feranil I. Inequities in family planning in low- and middle-income countries. *Glob Health Sci Pract*. 2023;11(3):e2300070. https://doi.org/10.9745/GHSP-D-23-00070

Supplement 2. The 69 Countries Included in the National Composite Index for Family Planning

**Asia**		**East and Southern sub-Saharan Africa**
Bangladesh		Botswana
Bhutan		Eswatini
Cambodia		Ethiopia
China		Gambia
India		Ghana
Indonesia		Kenya
Lao PDR		Lesotho
Mongolia		Liberia
Nepal		Malawi
Pakistan		Nigeria
Papua New Guinea		Sierra Leone
Philippines		Somalia
Timor-Leste		South Africa
Viet Nam		South Sudan
		Tanzania
**Eastern Europe, Central Asia**		Uganda
Armenia		Zambia
Azerbaijan		Zimbabwe
Georgia		
Kazakhstan		**Central and Western sub-Saharan Africa**
Kyrgyz Republic		Burkina Faso
Romania		Burundi
Tajikistan		Cameroon
Turkmenistan		Cote d'Ivoire
Uzbekistan		D.R. Congo
		Guinea
**Latin America and Caribbean**		Guinea-Bissau
Bolivia		Madagascar
Dominican Republic		Mali
Ecuador		Mozambique
El Salvador		Niger
Guatemala		Sao Tome and Principe
Haiti		Tchad
Honduras		Togo
Jamaica		
Peru		
**Middle East/North Africa**		
Djibouti		
Egypt		
Jordan		
Morocco		
Palestine		
